# Supplementary material for: Circulating Platelet-Derived Extracellular Vesicles Are a Hallmark of Sars-Cov-2 Infection
Source: Cells. 2021 Jan 7;10(1):85. doi: 10.3390/cells10010085 (PMC7825711; doi:10.3390/cells10010085)
Supplement: Supplementary file 1 [file cells-10-00085-s001.pdf]

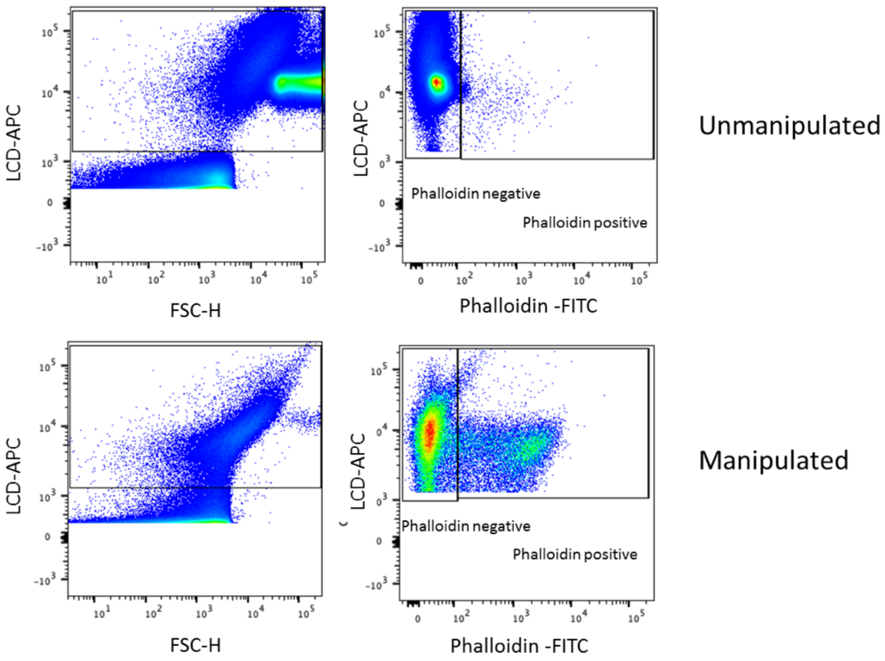

Supplementary Figure 1. Gating strategies showing the increase of phalloidin positive events resembling the presence of damaged PLT or, possibly, apoptotic bodies in manipulated blood sample.
